# Suppressive Effects of *Siegesbeckia orientalis* Ethanolic Extract on Proliferation and Migration of Hepatocellular Carcinoma Cells through Promoting Oxidative Stress, Apoptosis and Inflammatory Responses

**DOI:** 10.3390/ph15070826

**Published:** 2022-07-03

**Authors:** Tzu-Hua Chen, Chi-Chang Chang, Jer-Yiing Houng, Tzu-Hsien Chang, Ya-Ling Chen, Chia-Chang Hsu, Long-Sen Chang

**Affiliations:** 1 Institute of Biomedical Sciences, National Sun Yat-Sen University, Kaohsiung 80420, Taiwan; k59673@cgmh.org.tw; 2 Department of Nutritional Therapy, Kaohsiung Chang Gung Memorial Hospital, Kaohsiung 83340, Taiwan; 3 School of Medicine for International Students, College of Medicine, I-Shou University, Kaohsiung 82445, Taiwan; ed101779@edah.org.tw; 4 Department of Obstetrics & Gynecology, E-Da Hospital/E-Da Dachang Hospital, Kaohsiung 82445, Taiwan; ed112550@edah.org.tw (T.-H.C.); ed109776@edah.org.tw (Y.-L.C.); 5 Department of Nutrition, I-Shou University, Kaohsiung 82445, Taiwan; jyhoung@isu.edu.tw; 6 Department of Chemical Engineering, I-Shou University, Kaohsiung 82445, Taiwan; 7 School of Chinese Medicine for Post-Baccalaureate, College of Medicine, I-Shou University, Kaohsiung 82445, Taiwan; 8 Division of Gastroenterology and Hepatology, Department of Internal Medicine, E-Da Hospital, Kaohsiung 82445, Taiwan; 9 Health Examination Center, E-Da Dachang Hospital, Kaohsiung 80794, Taiwan

**Keywords:** *Siegesbeckia orientalis*, hepatocellular carcinoma, keratinocytes, proliferation and migration, oxidative stress, inflammatory response

## Abstract

Previous studies have demonstrated that *Siegesbeckia orientalis* (SO) has a suppressive effect on the growth and migration of endometrial and cervical cancer cells. The present study examined the effect of SO ethanolic extract (SOE) on the proliferation and migration of hepatocellular carcinoma (HCC) and examined the effects of SOE on non-cancerous cells using HaCaT keratinocytes as a model. The SOE effectively inhibited the proliferation of Hepa1-6 (IC_50_ = 282.4 μg/mL) and HepG2 (IC_50_ = 344.3 μg/mL) hepatoma cells, whereas it has less cytotoxic effect on HaCaT cells (IC_50_ = 892.4 μg/mL). The SOE treatment increased the generation of ROS in HCC, but decreased the expression of antioxidant enzymes such as superoxide dismutase, glutathione peroxidase and catalase. In contrast, it reduced intracellular ROS formation and upregulated the expression of the related antioxidant enzymes in the H_2_O_2_-stimulated HaCaT cells. The SOE intervention also down-regulated the anti-apoptotic Bcl-2 and the migration-related proteins including matrix metalloproteinases (MMPs) and β-catenin in the HCC, suggesting that SOE could promote HCC apoptosis and inhibit HCC migration. On the contrary, it reduced apoptosis and promoted the migration of the keratinocytes. Additionally, the SOE treatment significantly up-regulated the pro-inflammatory cytokines, including TNF-α, IL-6 and IL-1β, in Hepa1-6 and HepG2 cells. Conversely, it significantly decreased the expression of these cytokines in the H_2_O_2_-induced HaCaT cells. These findings indicated that SOE treatment can delay the progression of HCC by increasing oxidative stress, promoting inflammatory response, inducing cancer cell apoptosis and inhibiting their migration. It also has protective effects from pro-oxidant H_2_O_2_ in non-cancerous cells. Therefore, SOE may provide a potential treatment for liver cancer.

## 1. Introduction

Hepatocellular carcinoma (HCC) accounts for around 75–85% of primary liver cancer cases and is the fourth leading cause of cancer death worldwide [1,2,3]. Cirrhosis, alcohol abuse, chronic hepatitis B virus (HBV) or hepatitis C virus (HCV) infection, aflatoxin exposure and metabolic syndrome associated with diabetes and obesity are common causes of HCC [3,4]. For early-stage patients with HCC, the usual treatments are surgical resection and local ablation [5]. However, even in patients with a single tumor smaller than 2 cm, the recurrence rate of 5 years after hepatic resection is still as high as 70% [6].

When HCC progresses to the advanced stage, systemic therapy is usually employed, and sorafenib, which is a tyrosine kinase inhibitor approved by FDA, is an available standard treatment strategy [7]. Results from two large clinical trials have demonstrated that sorafenib was more effective than dozens of other molecules [8]. Unfortunately, the median overall survival of patients with advanced HCC receiving systemic therapy with this multi-kinase inhibitor is still only 1–2 years [3].

Hypoxia is one of the main features of solid tumors such as HCC. It renders the mitochondrial electron transport chain inefficient, resulting in elevated ROS (reactive oxygen species) levels and oxidative stress, thereby promoting carcinogenesis [7]. It is known that ROS can modify structural protein and is involved in cytoskeletal remodeling, drive cell migration and invasion through the formation of different types of cell protrusions such as filopodia, lamellipodia and invadopodia, and promote tumor cell invasion by stimulating the proteolytic degradation of ECM components [9]. Some studies have suggested that natural products with the ability to modulate ROS can promote the preferential killing of cancer cells [10]. For example, butein, a bioactive flavonoid isolated from plants, can suppress intracellular ROS production and induce cell cycle arrest and apoptosis and enhance caspase-3, -8 and -9 activity to inhibit cell viability, colony formation, migration and invasion of HeLa cells [11]. Kim et al. [12] showed that berberine, an isoquinoline alkaloid from Berberis species, increased AMPK activity to inhibit the metastatic potential caused by generating ROS. Han et al. [13] reported that the use of physcion isolated from both terrestrial and marine sources at lower cytotoxic concentrations could induce ROS generation, stimulate AMPK signaling, and inhibit cell adhesion, migration, and invasion in colorectal cancer cells.

It is generally accepted that ROS plays a tumor-promoting role in the early stage of tumorigenesis; whereas in the later stage of tumor progression, excess ROS can cause oxidative stress and lead to toxicity to cancer cells [8]. When ROS accumulate excessively, cancer cells in response, enhanced antioxidant systems, such as superoxide dismutase (SOD) and catalase, to counteract the ROS-generated cytotoxicity, thus inhibiting their antioxidant capacity in the later stage of tumor progression, have been proposed as an approach for cancer therapy [14,15]. Indeed, many chemotherapeutic agents that promote apoptotic or autophagic cell death are known to alter redox homeostasis in cancer cells by generating mitochondrial ROS and inhibiting cellular antioxidant systems [16,17]. For example, the chemotherapeutic drug cisplatin may induce mitochondria-dependent ROS, leading to cell death through the formation of nuclear DNA damage [18].

HCC is a highly drug-resistant cancer that requires adjuvant therapy [3]. Recently, several reports indicated that phytochemicals, such as resveratrol, ginsenoside Rg3 and silybin have been used for the prevention and adjuvant treatment of liver cancer [19,20,21,22]. 

Plants of the genus *Siegesbeckia,* belonging to the Asteraceae family, are widely distributed in tropical and temperate regions of the world [23]. *Siegesbeckia* *orientalis* (SO) has been documented to have therapeutic effects on wind-dampness, painful joints and bones, quadriplegia, traumatic bleeding and immune deficiency [24]. Recent literature reports that SO has anti-inflammation [25,26,27,28], anti-allergy [29], immunosuppression [29,30] anti-hyperuricemia [31], anti-diabetes [32,33], anti-bacterial [34,35] and anti-cancer properties [35,36,37,38,39], relieves rheumatoid arthritis [31,40] and knee osteoarthritis [41], attenuates postoperative cognitive dysfunction [42] and inhibits adipogenesis [43]. 

So far, there are several studies investigating the inhibitory effect of SO on cancer cell proliferation and metastasis [35,36,37,38,39]. The ethanolic extract of SO (SOE) has been shown to induce endometrial cancer (RL95-2) cell cycle arrest and cell apoptosis by up-regulating the proapoptotic genes, whereas down-regulating the antiapoptotic protein expression [36]. SOE has also been shown to inhibit migration and invasion of endometrial carcinoma (RL95-2 and HEC-1A) by decreasing the expression of migration-related genes, and the phosphorylation of ERK1/2, JNK1/2 and Akt [39].

This study investigated the inhibitory effect of SOE on the growth and migration of HepG2 and Hepa1-6 hepatoma cells, and compared with HaCaT non-cancerous keratinocytes, was stimulated by external oxidative stress with H_2_O_2_. At the same time, the effects of SOE intervention on intracellular oxidative stress, antioxidant system and inflammatory response were also examined to elucidate the bidirectional regulatory function of SOE on anti-hepatoma cells and protection of normal cells, which was not found in previous SOE anti-cancer literature. 

## 2. Results

### 2.1. Effect of SOE Treatment on Proliferation of Hepatoma Cells and Keratinocyte Cells

**Figure 1:**
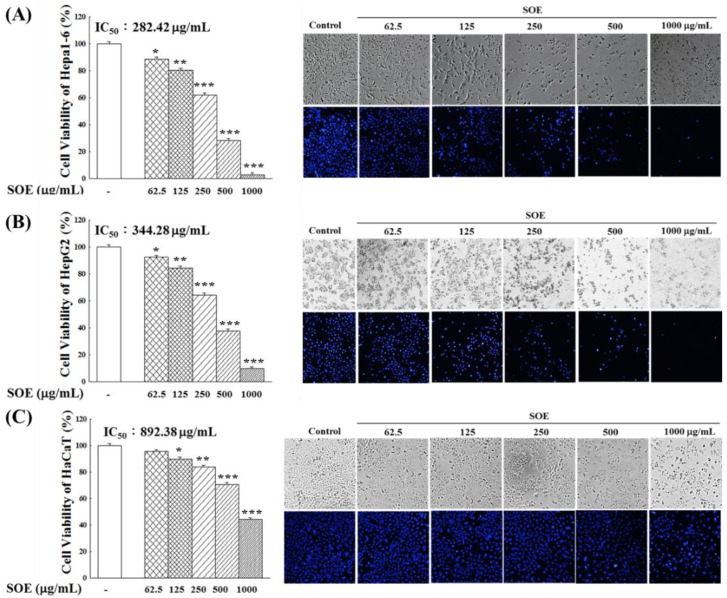
Cell viability and morphology of Hepa1-6 cells (**A**), HepG2 cells (**B**) and HaCaT cells (**C**), after treating with various concentrations of SOE for 72 h, as determined by MTT assay and DNA staining with Hoechst 33342 (blue). The control group was the cells without SOE intervention, and the number of cells cultured for 72 h was set as 100%. Levels of significance are expressed as * *p* < 0.05, ** *p* < 0.01 and *** *p* < 0.001.

Figure 1 shows the cell viability and morphology of Hepa1-6, HepG2 and HaCaT cells after the treatment of SOE for 72 h. The treatment of SOE suppressed the proliferation of all three cell lines in a dose-dependent fashion. However, SOE was more toxic to the liver cancer cells than to the HaCaT (non-cancerous cell). This is evident by the significantly lower IC_50_ values of Hepa1-6 cells (282.4 μg/mL) and HepG2 cells (344.3 μg/mL) in comparison with that of HaCaT cells (892.4 μg/mL). 

After treatment of Hepa1-6 and HepG2 liver cancer cells with SOE, results from the Hoechst 33342 cell nuclear staining analysis showed that the blue fluorescence expression decreased significantly with the increase of the SOE concentration, and in spite of their cell integrity and viability behaved similarly. However, HaCaT cells showed only a lesser degree of decrease in blue fluorescence at high SOE concentrations, indicating that SOE exhibited less cytotoxicity on HaCaT cells.

### 2.2. Effect of SOE Treatment on Oxidative System in Hepatoma and Keratinocyte Cells

#### 2.2.1. Viability of Keratinocyte Cells under H_2_O_2_ Stimulation

**Figure 2:**
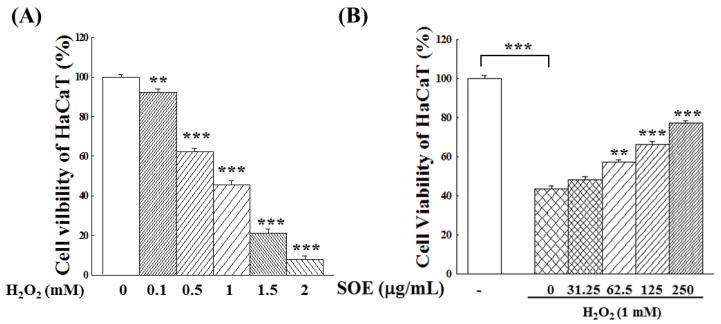
Effect of H_2_O_2_ and SOE on viability of HaCaT (non-cancerous cells). (**A**) Effect of H_2_O_2_-stimulation at different concentrations for 4 h on proliferation of HaCaT cells. The cells without H_2_O_2_ stimulation were used as the control group, and the number of cells was set as 100%. (**B**) Effect of SOE on viability of HaCaT cells subjected to 1 mM H_2_O_2_-stimulation for 4 h. The HaCaT cells were incubated with different concentrations of SOE for 72 h. After incubation, a fresh culture medium containing 1 mM H_2_O_2_ was added and incubated for an additional 4 h. Then, the cell viability was assessed by MTT assay. The HaCaT cells incubated without H_2_O_2_ induction and SOE treatment were used as the control, and its number of cells was set as 100%. The one-way ANOVA test was conducted to examine statistical differences between the vehicle (group 0) or between the control and the vehicle (Figure 2B). Levels of significance are expressed as ** *p* < 0.01 and *** *p* < 0.001.

To examine the effect of SOE treatment on oxidative system in liver cancer cells and keratinocytes, this study first investigated whether SOE treatment could affect intracellular ROS generation in these cells. However, unlike the two liver cancer cell lines, keratinocytes generated no significant amount of ROS during culture. Therefore, hydrogen peroxide (H_2_O_2_), an inducer for ROS generation was used to induce ROS formation in HaCaT cells. The survival rates of HaCaT cells after induction with different concentrations of H_2_O_2_ for 4 h were then examined. Results in Figure 2A demonstrate that the viability of HaCaT cells decreases significantly with the increase of H_2_O_2_ concentration, indicating that high H_2_O_2_ concentrations were cytotoxic to HaCaT cells. The concentration that caused half of the cell death (1 mM) was selected to compare the effect of SOE in subsequent experiments. 

Next, the effect of different concentrations of SOE on the proliferation of HaCaT cells for 72 h, followed by stimulation with 1 mM H_2_O_2_, was examined. As indicated by the results in Figure 2B, SOE attenuated the cell death induced by H_2_O_2_ stimulation. This protective effect of SOE was more pronounced and concentration-dependent.

#### 2.2.2. Intracellular ROS Changes

ROS play multiple roles in the development of HCC, so the effect of SOE treatment on intracellular ROS content was examined. As mentioned in the previous section, HaCaT cells produce only a small amount of ROS during culture, and H_2_O_2_ is known to induce ROS generation. In this study, cells were stimulated with 1 mM H_2_O_2_ to examine the influence of SOE on the intracellular ROS content in HaCaT cells. 

**Figure 3:**
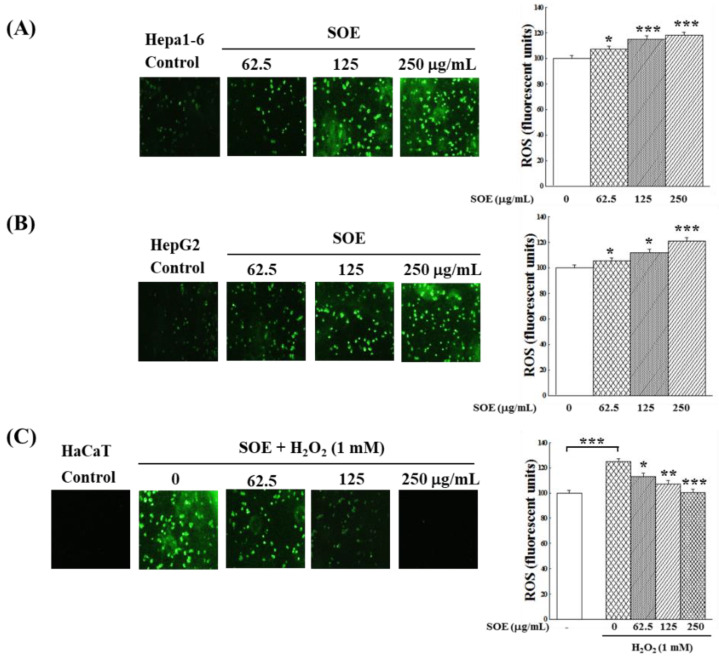
Effects of SOE on the changes of intracellular ROS content: (**A**) Hepa1-6 cells, (**B**) HepG2 cells and (**C**) H_2_O_2_-stimulated HaCaT cells. Hepa1-6 and HepG2 cells were cultivated for 24 h, various concentrations (0, 62.5, 125, 250 μg/mL) of SOE were then added. The cells were incubated for an additional 72 h, the levels of the intracellular ROS were monitored by DCFH_2_-DA fluorescence intensity. For HaCaT cells, cultivated the cells for 24 h, then various concentrations (0, 62.5, 125, 250 μg/mL) of SOE were added. After an additional 72 h cultivation, the cells were changed to a fresh culture medium containing 1 mM H_2_O_2_ and incubated for another 4 h. Then, the intracellular ROS levels were detected by the DCFH_2_-DA fluorescence. The control group was the HaCaT cells incubated without H_2_O_2_ induction and SOE treatment, and its ROS amount was set as 100%. The one-way ANOVA test was conducted to examine statistical differences between the vehicle (group 0) or between the control and the vehicle (Figure 3C). Levels of significance are expressed as * *p* < 0.05, ** *p* < 0.01 and *** *p* < 0.001.

Figure 3A,B show that SOE treatment increased the generation of ROS in Hepa1-6 and HepG2 cells in a dose-dependent pattern. This finding indicates that SOE treatment decreasing the viability of Hepa1-6 and HepG2 cells (Figure 1) was partly a result of an increase in ROS level. However, Figure 3C shows that the expression of ROS was significantly increased when HaCaT cells were stimulated by H_2_O_2_. SOE treatment reduced the ROS content in H_2_O_2_-stimulated HaCaT cells, and when SOE concentration increased to 250 μg/mL, the level of ROS generated by the HaCaT cells dropped to the level of the control group. 

#### 2.2.3. Expression of Antioxidant Enzymes

**Figure 4:**
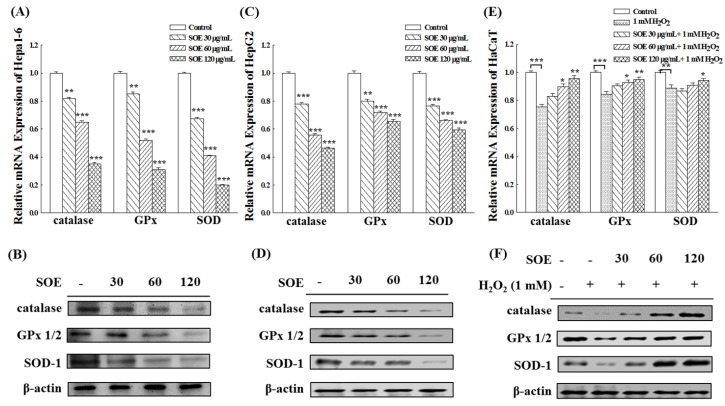
Effect of SOE treatment on expression of antioxidant enzymes in Hepa1-6 cells (**A**,**B**), HepG2 cells (**C**,**D**), and H_2_O_2_–stimulated HaCaT cells (**E**,**F**). Levels of significance are expressed as * *p* < 0.05, ** *p* < 0.01 and *** *p* < 0.001.

The effect of treatment with various concentrations of SOE (0, 30, 60, 120 μg/mL) on gene and protein expression of three different antioxidant enzymes, including catalase, glutathione peroxidase (GPx) and superoxide dismutase (SOD), in liver cancer cells (Hepa1-6 and HepG2) and non-cancerous cells (HaCaT stimulated with H_2_O_2_) were examined. Figure 4A–D shows that the genes and protein expression of these three enzymes levels in both Hepa1-6 and HepG2 cells decreased as SOE concentration increased. The effects were more pronounced on Hepa1-6 cells (Figure 4A,B) than HepG2 cells (Figure 4C,D). 

In terms of HaCaT cells, when HaCaT cells were stimulated by H_2_O_2_, the expression of these three enzymes decreased significantly. However, the production of these three enzymes increased when H_2_O_2_-stimulated HaCaT cells were treated with SOE, more so when the SOE dosage increased (Figure 4E,F). The results of Western blot analysis (Figure 4F) show the protein expression levels of these three enzymes were restored to normal levels when the SOE concentrations were 60 and 120 μg/mL.

### 2.3. Effect of SOE on the Expression of Anti-Apoptotic Bcl-2 Gene and Protein

**Figure 5:**
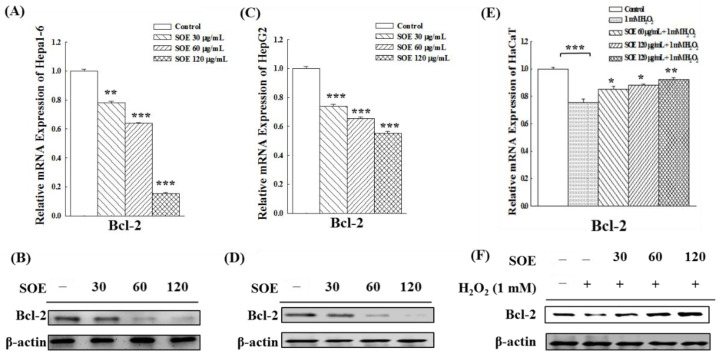
Effect of SOE treatment on expression of the Bcl-2 gene and protein in Hepa1-6 cells (**A**,**B**), HepG2 cells (**C**,**D**), and H_2_O_2_–stimulated HaCaT cells (**E**,**F**). Levels of significance are expressed as * *p* < 0.05, ** *p* < 0.01 and *** *p* < 0.001.

Bcl-2 gene is an important apoptosis inhibitory gene [44]. To determine how SOE affects the apoptosis of cell lines (Hepa1-6, HepG2 and HaCaT), the expression levels of the Bcl-2 gene and protein were measured. Results show that SOE treatment significantly decreased both Bcl-2 mRNA expression levels and protein levels in Hepa1-6 (Figure 5A,B) and HepG2 (Figure 5C,D) cells. The change showed a dose-dependent fashion, indicating that SOE promoted the apoptosis in these two liver cancer cell lines. 

For non-cancerous keratinocytes cells (HaCaT), the expression of Bcl-2 gene and protein were significantly decreased under the H_2_O_2_ stimulation (Figure 5E,F), indicating that H_2_O_2_-induction led to apoptosis in HaCaT cells. However, the expression of Bcl-2 was significantly increased after SOE treatment, suggesting that SOE could protect HaCaT cells from apoptosis induced by the pro-oxidant H_2_O_2_.

### 2.4. Effects of SOE on Migration of Hepatoma and Keratinocyte Cells

#### 2.4.1. Wound Healing Assay

**Figure 6:**
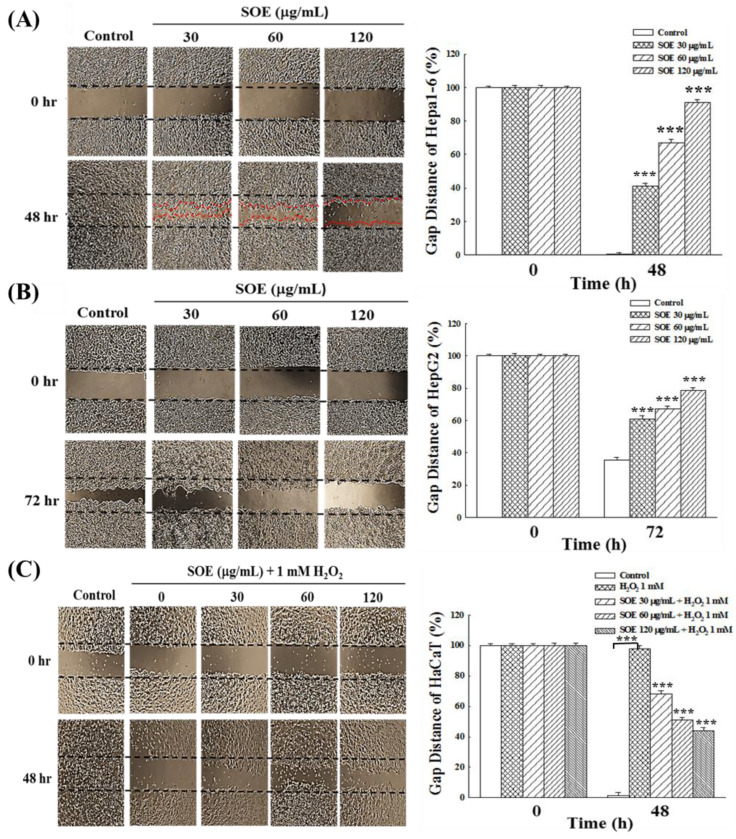
Effect of SOE on cell migration examined by wound healing assay: (**A**) Hepa1-6 cells, (**B**) HepG2 cells, and (**C**) H_2_O_2_-stimulated HaCaT cells. Cell lines were seeded into culture dishes with migration inserts in 6 cm dishes and cultivated for 24 h. Then, the inserts were removed, and the indicated concentration of SOE was added. For HaCaT cells, 1 mM H_2_O_2_ was also added into the culture medium. The wounded areas for Hepa1-6 and HaCaT cells at 0 and 48 h, and HepG2 cells at 0 and 72 h for HepG2 cells were photographed. The effect on cell migration is expressed by gap distance. Levels of significance are expressed as *** *p* < 0.001.

For wound repair and tumor metastasis, cells typically migrate collectively through tight cell-cell junctions, also known as collective migration [45]. Figure 6 presents the effects of SOE treatment on the wound healing of HCCs (Hepa1-6 and HepG2 cells) and non-cancerous cells (HaCaT). The reason that the observation time was 48 h for Hepa1-6 cell line, but 72 h for HepG2 cells, was due to the slower moving speed of HepG2 cells. Without SOE treatment, both hepatoma cell lines migrated rapidly to the scratched area and their cell numbers were also increased rapidly over time. When both hepatoma cell lines were treated with SOE, the ability of hepatoma cells to migrate was reduced in a concentration-dependent manner (Figure 6A,B). When Hepa1-6 and HepG2 cells were treated with SOE at 120 μg/mL for 48 and 72 h, the percentage of gap distance Hepa1-6 and HepG2 cells was found to be 90.2 ± 1.6% and 78.6 ± 1.8%, respectively.

Regarding HaCaT cells, cells in the control not subjected to oxidative stress had intact intercellular junctions (the control in Figure 6C), whereas in cells stimulated with H_2_O_2_, the cell-to-cell junctions were inhibited (the vehicle (group 0) in Figure 6C). However, when treating the H_2_O_2_-stimulated cells with SOE, this inhibitory effect was blocked, and the ability of cell migration increased with the increase of SOE concentration. After 48 h treatment with 120 μg/mL SOE, the percentage of gap distance was reduced to 44.2 ± 2.1%.

The above results illustrate that SOE treatment can significantly reduce the migration ability of liver cancer cells; on the other hand, SOE can alleviate the H_2_O_2_-inhibitory effect on non-cancerous cells (HaCaT) and promote cell migration and thus help wound healing.

#### 2.4.2. Expression of Migration-Related Proteins

**Figure 7:**
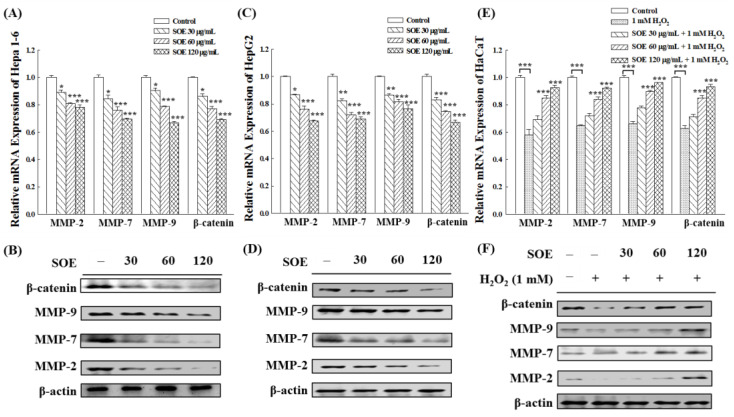
Effect of SOE treatment on expression of the migration-related genes and proteins in Hepa1-6 cells (**A**,**B**), HepG2 cells (**C**,**D**), and H_2_O_2_-stimulated HaCaT cells (**E**,**F**). Levels of significance are expressed as * *p* < 0.05, ** *p* < 0.01 and *** *p* < 0.001.

It is known that β-catenin and MMPs are involved in multiple regulatory processes related to cell migration and invasion [46,47,48]. Thus, the gene and protein expression of β-catenin, MMP-2/7/9 in HCCs (Hepa1-6, HepG2 cells) and non-cancerous cells (HaCaT) were examined by RT-qPCR and Western blot analysis. Figure 7A–D show that SOE intervention significantly reduced the genes and proteins levels of β-catenin, MMP-2/7/9 in Hepa1-6 and HepG2 cells. On the other hand, SOE functioned differently on HaCaT cells. The expressions of β-catenin and MMP-2/7/9 in HaCaT cells were significantly decreased under the stimulation of H_2_O_2_. When the H_2_O_2_-atimulated HaCaT cells were treated with SOE, the expression of β-catenin, MMP-2/7/9 was up-regulated (Figure 7E,F).

### 2.5. Effects of SOE on Inflammation in Hepatoma and Keratinocyte Cells

**Figure 8:**
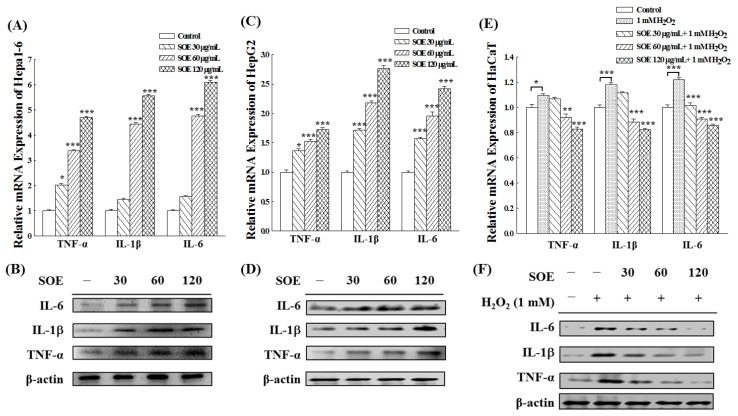
Expression of the genes and proteins of the pro-inflammatory cytokines (IL-6, IL-1β and TNF-α) under the treatment of SOE in Hepa1-6 cells (**A**,**B**), HepG2 cells (**C**,**D**), and H_2_O_2_-stimulated HaCaT cells (**E**,**F**) for 72 h cultivation. Levels of significance are expressed as * *p* < 0.05, ** *p* < 0.01 and *** *p* < 0.001.

Figure 8 shows the effect of SOE treatment on the gene and protein expression of pro-inflammatory cytokines in two hepatoma cell lines (Hepa1-6 and Hepa1-6) and non-cancerous cells (HaCaT). Figure 8A–D show that SOE treatment significantly up-regulated the gene and protein expression of the pro-inflammatory cytokines, including IL-6, IL-1β and TNF-α, in Hepa1-6 and HepG2 cells. This indicates that SOE treatment induces acute inflammation in HCC, and the degree of inflammation increased with the increase of SOE treatment concentration.

For HaCaT cells, the expression of these three pro-inflammatory cytokines (IL-6, IL-1β and TNF-α) was significantly enhanced under H_2_O_2_ stimulation (as shown in Figure 8E,F), indicating that H_2_O_2_-induction caused acute inflammation in HaCaT cells. However, the expression of these three cytokines was significantly decreased when treated with SOE, which indicates that SOE exhibited an anti-inflammatory effect on the H_2_O_2_-stimulated HaCaT cells.

## 3. Discussion

Primary liver cancer is the most frequently diagnosed cancer and the sixth leading cause of cancer death worldwide, with HCC accounting for 75–85% of primary liver cancers [2]. HCC is a highly drug-resistant cancer that requires adjuvant therapy [3]. Plants of *Siegesbeckiae* have been documented to have the therapeutic effects on wind-dampness, painful joints and bones, quadriplegia, traumatic bleeding and immune deficiency [24]. Several studies have investigated the inhibitory effect of SO on cancer cell proliferation and metastasis [35,36,37,38,39].

In this study, cell viability and Hoechst 33,342 cell nuclear staining showed significantly low in the HCCs (Hepa1-6, HepG2 cells) with treatment of SOE and were significantly lower than those in non-cancerous keratinocytes cells (HaCaT), indicating that SOE exhibited less cytotoxicity on HaCaT cells (Figure 1).

Chuang et al. [49] have previously reported that H_2_O_2_ could generate lots of free radicals such as OH, thereby inducing apoptosis of cardiomyocyte HL-1 cells through intrinsic or mitochondrial pathways. Sun et al. [50] have shown that H_2_O_2_induced oxidative damage in HaCaT cells, resulting in decreased cell viability and increased apoptosis rate. In this study, results in Figure 2A confirmed the findings of Sun et al. [50] that the viability of HaCaT (non-cancerous cell) decreases significantly with the increase of H_2_O_2_ concentration, indicating that high H_2_O_2_ concentrations were cytotoxic to HaCaT cells. Experimental results shown in Figure 2B demonstrate that SOE enhanced the survival of HaCaT from the toxicity of the pro-oxidant H_2_O_2_.

ROS play multiple roles in the development of HCC, so the effect of SOE treatment on intracellular ROS content was examined. Figure 3 shows that SOE treatment increased the generation of ROS in Hepa1-6 and HepG2 cells in a dose-dependent pattern, and this finding indicates that SOE treatment decreased the viability of Hepa1-6 and HepG2 cells (Figure 1) in part due to elevated ROS levels. Previously, Chen et al. [51] have reported that treatment of HCCs (HepG2 and Huh7 cells) with piperlongumine, a natural product isolated from longer capsicum plants, significantly increased the level of ROS in cells, thereby exerting an anticancer effect on HCC. Results in Figure 3 have also shown that SOE treatment reduced the ROS content in H_2_O_2_-stimulated HaCaT cells, which suggested the increased viability of SOE-treated HaCaT cells (Figure 2) was due to a reducing ROS production. This finding is in line with that reported by Sakan et al., that the use of dendropachol, a phytochemical isolated from *Dendrobium officinale*, protected the H_2_O_2_-stimulated HaCaT cells by inhibiting the production of intracellular ROS [52].

SOE has been shown to have good in vitro antioxidant activities, including radical-scavenging capacity on DPPH (IC_50_ = 161.8 µg/mL) and ABTS (IC_50_ = 13.9 µg/mL), and reducing power [32]. In addition, for the oxidative damage to pancreatic β-cells when exposed to a high glucose-stimulated glucotoxic environment, the SOE treatment could effectively reduce the production of ROS, increase the content of intracellular glutathione, up-regulate the expression of antioxidant enzymes and enhance the survival rate of β-cells. Therefore, SOE has a protective effect on non-cancerous pancreatic β-cells under the high glucose-induced conditions [33]. In this study, the genes and proteins expression levels of catalase, glutathione peroxidase (GPx) and superoxide dismutase (SOD) were significantly decreased in HaCaT (non-cancerous cells) under H_2_O_2_ stimulation. The values of these enzymes returned to normal levels after the addition of SOE (Figure 4). However, SOE has an opposite regulatory effect on oxidative stress in liver cancer cells, increasing intracellular ROS and downregulating the expression of antioxidant enzymes in cancer cells. Figure 4 shows that the gene and protein expression levels of catalase, glutathione peroxidase (GPx) and superoxide dismutase (SOD) decreased in HCCs (Hepa1-6 and HepG2 cells) with increasing SOE concentration. This effect may have contributed to the apoptotic effect of SOE on hepatoma cells [16,17].

In intrinsic apoptosis, irreversible mitochondrial outer membrane permeabilization (MOMP) is controlled by members of the Bcl-2 protein family which function as pro-apoptotic and anti-apoptotic [53]. Among them, the Bcl-2 gene is an important apoptosis inhibitory gene. Bcl-2 can block the transfer of cytochrome C from mitochondria to cytoplasm and inhibit the activation of caspase, thereby inhibiting apoptosis [44]. The results in Figure 5 show that SOE treatment significantly decreased the Bcl-2 gene and protein expression level in Hepa1-6 and HepG2 cells in a dose-dependent fashion, indicating that SOE promoted apoptosis in these two liver cancer cell lines. Previous studies have elucidated that SOE inhibited the growth of RL95-2 human endometrial cancer cells through the intrinsic and extrinsic apoptotic pathways [36]. SOE treatment up-regulated the expression of caspase-3, -8, -9, Bad, Bak and Bax, decreased the protein expression of Bcl-2 and Bcl-xL and resulted in cell cycle arrest in the G2/M phase [36]. In this study, HaCaT (non-cancerous cells), when stimulated with H_2_O_2_, revealed that the expression of the Bcl-2 gene and protein were decreased (Figure 5). This finding is consistent with several papers asserting that induction of oxidative damage by H_2_O_2_ can promote apoptosis in HaCaT cells, because it increases the expression and activation of pro-apoptotic proteins and reduces the expression of the anti-apoptotic protein [54,55,56]. However, when the H_2_O_2_-stimulated HaCaT cells were treated with SOE, the expression of the Bcl-2 was significantly increased in a dose-related fashion (Figure 5). The results of this study indicate that SOE can protect HaCaT cells from apoptosis induced by the pro-oxidant H_2_O_2_.

For wound repair and tumor metastasis, cells typically migrate collectively through tight cell-cell junctions, also known as collective migration [45]. The tumor metastasis cascade is a multi-step process involving local tumor cell invasion and migration to distant sites [57]. In this study, the wound healing assay results showed that SOE treatment can significantly reduce the migration ability of liver cancer cells (Hepa1-6 and HepG2 cells); on the other hand, SOE can alleviate the H_2_O_2_-inhibitory effect on HaCaT (non-cancerous cells) and promote cell migration and thus help wound healing (Figure 6).

It is known that β-catenin and MMPs are involved in multiple regulatory processes related to cell migration and invasion [46,47,48]. Cell metastasis involves complex processes, including degradation of the extracellular cell matrix (ECM), separation of cells, adhesion of cells to endothelial cells, cell migration, cell invasion, cell motility and rebuilding of the growing system at a distance site [58]. Through the above processes, the degradation of the ECM is the key step in cell migration and invasion, a biochemical and biophysical barrier to cell migration and invasion and a major phenotype of cancer metastasis [59]. Therefore, targeting the EMT process is considered an excellent strategy to prevent cancer metastasis [60,61,62,63]. Studies have shown many proteolytic enzymes that are responsible for ECM degradation. Among them, matrix metalloproteinases (MMPs) are critical in ECM degradation associated with tumor cell migration, invasion and angiogenesis. The MMPs can regulate tumor growth, tissue remodeling, inflammation, invasion and metastasis. Hence, MMPs inhibitors have the potential as chemo-preventive agents against cancer cells [54]. Among the family of MMPs, activated MMP-2, -7, -9, -12 can be converted into active plasmin to degrade plasminogen, including loss of cell–cell adhesion and increased cell mobility, whereas MMP-2 and -9 are associated with blood vessel formation [64]. MMP-2, -7 and -9 have been confirmed to play important roles in migration and invasion of liver cancer cells [65,66,67,68]. They also have important impact on the migration of HaCaT cells [69,70,71]. 

The Wnt/β-catenin signaling pathway is essential in embryonic development, angiogenesis, stem cell differentiation and self-renewal of adult tissues. When abnormally activated, this pathway leads to abnormal cell proliferation and malignant transformation. β-Catenin is a core component of the Wnt signaling pathway, regulating the transcription of several downstream target genes of Wnt, such as MMPs, c-myc, cyclin D1 and vimentin, thereby mediating cell proliferation, apoptosis, metastasis and invasion [46,47,48]. According to statistics, approximately 30% of HCC patients have abnormally high levels of Wnt/β-catenin signaling pathway activity [48,63,72,73]. In HCC, inhibition of expression or activity of β-catenin decreases migration and invasion of cancer cells [60,63]. Furthermore, the Wnt/β-catenin signaling pathway is also critical in wound healing. Much evidence has shown that activation of the Wnt/β-catenin signaling pathway can promote cell proliferation and migration during wound healing. β-Catenin acts as an integral component in this signaling pathway and plays a regulatory role [74,75,76,77]. 

Previous study has shown that SOE inhibited dose-dependently the expression of MMP-9, MMP-2 and u-PA in endometrial cancer RL95-2 cells, thereby inhibiting migration and invasion [39]. In this study, SOE treatment of HCCs (Hepa1-6, HepG2) showed that the genes and proteins levels of β-catenin, MMP-2/7/9 were significantly reduced by RT-qPCR and Western blot analysis. In addition, the genes and proteins expression levels of β-catenin, MMP-2/7/9 were significantly decreased in HaCaT (non-cancerous cell) under H_2_O_2_ stimulation. The values of these proteins returned to normal levels after the addition of SOE (Figure 7). The findings that SOE possessed the capability of inhibiting HCCs (Hepa1-6, HepG2) migration and invasion and promote non-cancerous keratinocyte (HaCaT) wound healing are consistent with their opposing effects on cell migration (Figure 6). These results illustrate that SOE can modulate the Wnt/β-catenin signaling pathway, and inhibit the migration and invasion of hepatoma cells. On the other hand, it can promote the cell–cell junctions of non-cancerous keratinocytes under H_2_O_2_ oxidative stress.

Cytokines including pro- and anti-inflammatory cytokines released by inflammatory cells are major signaling molecules. Generally, the effect of inflammation on most cancers is bidirectional. Induction of acute inflammatory responses in cancer therapy would stimulate dendritic cell maturation and antigen presentation, leading to antitumor immune responses that kill cancer cells [78,79,80]. Figure 8 shows that SOE treatment significantly up-regulated the gene and protein expression of the pro-inflammatory cytokines (including IL-6, IL-1β and TNF-α) in Hepa1-6 and HepG2 cells, indicating that SOE treatment can induce acute inflammation in HCCs. On the other hand, during cancer development, IL-1α and IL-1β in chronic inflammation can contribute directly to the production of oncogenic mediators such as nitric oxide and ROS, and excessive IL-6 and IL-11 binding to oncogenic driver mutations leads to overactivation of STAT3 and the development of malignancy. In other words, chronic inflammation provides a good microenvironment for tumor initiation, development and metastasis [81,82]. Therefore, in cancer therapy, treatment-induced chronic inflammation would promote tumor progression and treatment resistance. For non-cancerous cells (HaCaT), the expression of these three pro-inflammatory cytokines (IL-6, IL-1β and TNF-α) was significantly enhanced under the H_2_O_2_ stimulation (as shown in Figure 8), indicating that H_2_O_2_-induction caused acute inflammation in HaCaT cells. However, the expression of these three cytokines was significantly decreased when treated with SOE, which indicates that SOE exhibited an anti-inflammatory effect on the H_2_O_2_-stimulated HaCaT cells. Previously, Hong et al. [25] have demonstrated that SOE has an anti-inflammatory effect against acute inflammation in a mouse model induced by subcutaneous injection of λ-carrageenan or intraperitoneal injection of lipopolysaccharide (LPS), as well as in LPS-stimulated murine macrophage RAW264.7 cells. They also showed that SOE attenuated acute inflammation by suppressing inflammatory mediators through MAPKs- and NF-κB-dependent pathways. The present study demonstrated that SOE has the anti-inflammatory activity in HaCaT stimulated by H_2_O_2_. To our best knowledge, this is the first report demonstrating the pro-inflammatory effects of SOE on HCC.

## 4. Materials and Methods

### 4.1. Preparation of SOE

SO was bought from Yuanshan Herbal Shop (Kaohsiung City, Taiwan). The nucleotide sequence of this plant has been registered in the NCBI database with accession number of JN987228 [83]. The extraction procedure and analysis of chemical composition in SOE have been reported in our previous paper [33]. Briefly, SOE was obtained by extracting the dry aerial part of SO with 95% ethanol for 24 h and repeated 3 times, followed by concentration under reduced pressure and freeze-drying. The extraction yield was 5.26%. The dried SOE was kept at −20 °C before use.

### 4.2. Cell Culture and Viability Analysis

The cell lines of Hepa1-6 murine hepatoma cell, HepG2 human hepatoma cell and HaCaT human keratinocytes were purchased from Bioresource Collection and Research Center (Hsinchu, Taiwan). These cell lines were incubated at 37 °C, 5% CO_2_, 95% air (humidified incubator). The medium was Dulbecco’s Modified Eagle Medium (DMEM) supplemented with 10% fetal bovine serum (FBS) (Gibco Co., Grand Island, NY, USA), 100 U/mL penicillin-100 μg/mL streptomycin and 1% L-glutamine, 0.02% NaHCO_3_, pH 7.2–7.4. Cells at a density of 5 × 10^4^ cells/mL were cultured in 96-well plates for 24 h, washed three times with PBS and then cultured for 72 h with the addition of SOE at the indicated concentrations. For the cell viability assay, the culture medium was removed, and cells were washed 3 times with PBS and 100 µL MTT (5 mg/mL) added. After incubation for 2 more hours, the medium solution was replaced with 100 µL of dimethyl sulfoxide (DMSO) and the plate was shaken until all the crystals were dissolved. The cell viability was detected at wavelength of 570 nm in an ELISA reader (Model 550, Bio-Rad Laboratories, Hercules, CA, USA).

### 4.3. Assay of Intracellular ROS Level

Intracellular ROS levels were detected with 2’,7’-dichlorodihydrofluorescein diacetate (DCF-DA) by the dichlorofluorescein assay kit (Sigma-Aldrich Chemicals, St. Louis, MO, USA). Cells (Hepa1-6, HepG2 and HaCaT) were cultured at a density of 5 × 10^4^ cells/mL in 96-well dishes at 37 °C, 5% CO_2_ for 24 h. The medium was removed, cells were washed with PBS and incubated with various SOE concentrations for 72 h. For HaCaT keratinocytes, the cells were further stimulated by adding 1 mM H_2_O_2_ and incubated for additional 4 h. After incubation, the medium was removed, cells were washed with PBS and 100 μL of 5 μM DCF-DA was added and allowed to stand at room temperature for 1 h. The ROS level was then detected by fluorescence in a microplate reader with excitation at 502 nm and emission at 524 nm (Synergy^TM^ 2, BioTek, Winooski, VT, USA).

### 4.4. Gene Expression Analysis with Quantitative RT-PCR

The number of 5 × 10^4^ cells/mL was cultured in a 96-well culture plate for 24 h, Then, we replaced the medium with the culture medium containing different concentrations of SOE and continued to culture for 72 h. For HaCaT keratinocytes, cells were further stimulated by adding 1 mM H_2_O_2_ and incubated for additional 4 h. The washed cells were collected by centrifugation (1200 rpm, 5 min). RNA was extracted from cells using Qiagen RNeasy kit (Qiagen, Venlo, The Netherlands) and reverse transcribed to cDNA by using the Magic RT cDNA synthesis kit (Bio-Genesis, Taipei, Taiwan). The cDNA fragments were amplified with the IQ2 SYBR Green Fast qPCR Synthesis Master Mix LOW ROX Kit (Bio-Genesis) and Fast Dx Real-Time PCR Instrument (Model 7500, Applied Biosystems, Foster City, CA, USA). The qRT-PCR reaction was conducted with the first stage at 50 °C for 2 min, the second stage at 95 °C for 10 min and the third stage at 95 °C for 15 s and 60 °C for 1 min. A total of 40 cycles were performed. The primers used for qRT-PCR are listed in Table 1. The β-actin mRNA was used as an internal control. The obtained data were analyzed by iQ5 Optical System Software (Version 2.0, Bio-Rad).

### 4.5. Western Blot Assay

Western blot was used to detect the protein expression of antioxidant enzymes, MMPs, Bcl-2, inflammatory cytokines and the internal standard of β-actin. The assay mainly followed the procedures described previously [36,39]. All the antibodies used were bought from Sigma-Aldrich. The specific proteins were probed with their primary antibodies (1:5000 dilution). The membranes were hybridized with the horseradish peroxidase-conjugated secondary antibody (1:2000 dilution). The expression of proteins was detected and analyzed by the ChemiDoc XRS+ System (Bio-Rad).

### 4.6. Cell Migration by Wound Healing Assay

The culture dish containing 1 × 10^6^ cells and a migration insert (Sigma-Aldrich) was incubated at 37 °C for 24 h. Then, the insert was removed and the culture medium containing different concentrations of SOE was added. For HaCaT cells, 1 mM H_2_O_2_ was also added into the culture medium. We photographed the wounded areas with an inverted phase-contrast microscope (Eclipse TS100, Nikon Instruments, Tokyo, Japan), at 0 and 48 h for Hepa1-6 and HaCaT cells, and at 0 and 72 h for HepG2 cells. The cell migration is expressed as the level of gap distance.

### 4.7. Statistical Analysis

Five replicates were performed for each experiment, and the data were denoted as the mean ± standard deviation (SD). The statistical differences were analyzed by the one-way ANOVA test using the Microsoft Excel software (Office 2019, Microsoft Software Inc., Redmond, WA, USA). Levels of significance were indicated as * *p* < 0.05, ** *p* < 0.01, and *** *p* < 0.001.

## 5. Conclusions

The evidence provided by this study indicates that SOE possesses the inhibitory effect on proliferation and migration of HCC. The effect is mediated by promoting oxidative stress, stimulating acute inflammatory response, inducing cancer cell apoptosis and inhibiting cell proliferation and migration. Although SOE has shown a low cytotoxicity on HaCaT cells, SOE, nevertheless, can enhance the activity of the intracellular antioxidant system, reduce cell apoptosis, promote cell migration and reduce inflammation to protect these non-cancerous cells stimulated by external oxidative stress. Therefore, SOE has the potential to be used as an adjuvant therapy for liver cancer.

## Figures and Tables

**Table 1:** The primers applied in qRT-PCR assay.

Primer	Sequence
Catalase	5′-GCCATTGCCACAGGAAAGTA-3′
	5′-CCTTGGTGAGATCGAATGGA-3′
GPx	5′-CCAAGCTCATCACCTGGTCT-3′
	5′-TCGATGTCAATGGTCTGGAA-3′
SOD	5′-TGGCCGATGTGTCTATTGAA-3′
	5′-CACCTTTGCCCAAGTCATCT-3′
Bcl-2	5′-CTGAGTACCTGAACCGGCA-3′
	5′-GAGAAATCAAACAGAGGCCG-3′
β-Catenin	5′-ATTGATTCGAAACCTTGCCC-3′
	5′-AGCTCCAGTACACCCTTCTA-3′
MMP-2	5′-AGAACTTCCGATTATCCCATGATGA-3′
	5′-TGACAGGTCCCAGTGTTGGTG-3′
MMP-7	5′-GGCGGAGATGCTCACTTTGAC-3′
	5′-AATTCATGGGTGGCAGCAAAC-3′
MMP-9	5′-GCCCTGGAACTCACACGACA-3′
	5′-TTGGAAACTCACACGCCAGAAG-3′
IL-6	5′-TGGAGTACCATAGCTACCTGGAGT-3′
	5′-TCCTTAGCCACTCCTTCTGTGACT-3′
IL-1β	5′-GGTCAAAGGTTTGGAAGCAG-3′
	5′-TGTGAAATGCCACCTTTTGA-3′
TNF-α	5′-CAGGTTCTGTCCCTTTCACTCACT-3′
	5′-GTTCAGTAGACAGAAGAGCGTGGT-3′
GAPDH	5′-TGCACCACCAACTGCTTAGC-3′
	5′-GGCATGGACTGTGGTCATGAG-3′

## Data Availability

Data is contained within the article.
